# Short-term, high-fat diet accelerates disuse atrophy and protein degradation in a muscle-specific manner in mice

**DOI:** 10.1186/s12986-015-0037-y

**Published:** 2015-11-04

**Authors:** Steven L. Roseno, Patrick R. Davis, Lance M. Bollinger, Jonathan J. S. Powell, Carol A. Witczak, Jeffrey J. Brault

**Affiliations:** East Carolina Diabetes and Obesity Institute, East Carolina University, Greenville, NC USA; Human Performance Lab, Department of Kinesiology, College of Health and Human Performance, East Carolina University, Greenville, NC USA; Department of Kinesiology and Health Promotion, College of Education, University of Kentucky, Lexington, KY USA; Department of Physiology, Brody School of Medicine, East Carolina University, Greenville, 27834 NC USA; Department of Biochemistry and Molecular Biology, Brody School of Medicine, East Carolina University, Greenville, NC USA

**Keywords:** Obesity, Skeletal muscle, Muscular atrophy

## Abstract

**Background:**

A short-term high-fat diet impairs mitochondrial function and the ability of skeletal muscle to respond to growth stimuli, but it is unknown whether such a diet alters the ability to respond to atrophy signals. The purpose of this study was to determine whether rapid weigh gain induced by a high-fat (HF) diet accelerates denervation-induced muscle atrophy.

**Methods:**

Adult, male mice (C57BL/6) were fed a control or HF (60 % calories as fat) diet for 3 weeks (3wHF). Sciatic nerve was sectioned unilaterally for the final 5 or 14 days of the diet. Soleus and extensor digitorum longus (EDL) muscles were removed and incubated in vitro to determine rates of protein degradation and subsequently homogenized for determination of protein levels of LC3, ubiquitination, myosin heavy chain (MHC) distribution, and mitochondrial subunits.

**Results:**

When mice were fed the 3wHF diet, whole-body fat mass more than doubled, but basal (innervated) muscle weights, rates of protein degradation, LC3 content, mitochondrial protein content, and myosin isoform distribution were not significantly different than with the control diet in either soleus or EDL. However in the 14 day denervated soleus, the 3wHF diet significantly augmented loss of mass, proteolysis rate, amount of the autophagosome marker LC3 II, and the amount of overall ubiquitination as compared to the control fed mice. On the contrary, the 3wHF diet had no significant effect in the EDL on amount of mass loss, proteolysis rate, LC3 levels, or ubiquitination. Fourteen days denervation also induced a loss of mitochondrial proteins in the soleus but not the EDL, regardless of the diet.

**Conclusions:**

Taken together, a short-term, high-fat diet augments denervation muscle atrophy by induction of protein degradation in the mitochondria-rich soleus but not in the glycolytic EDL. These findings suggest that the denervation-induced loss of mitochondria and HF diet-induced impairment of mitochondrial function may combine to promote skeletal muscle atrophy.

## Background

Loss of muscle mass and strength increase disability [1] and are independent predictors of mortality [2, 3]. The deleterious effects of low muscle mass are compounded during obesity, as obese individuals with less muscle are at a greater risk for death due to certain cancers [4, 5], cardiovascular disease [6, 7], and renal disease [8, 9]. Furthermore, severely obese individuals tend to be much less active, and muscle disuse is a potent inducer of muscle mass loss [10]. Despite the connection between weight gain, low muscle mass, and increased mortality, little is known about how obesity influences skeletal muscle atrophy.

The duration of hypercaloric feeding seems to have an effect on protein metabolism. Long-term (5 month) high-fat feeding of C57BL/6 mice [11] or life-long genetic mutation of the leptin receptor (*db/db*) [12] leads to severe obesity, increased basal rate of muscle protein degradation, and loss of skeletal muscle mass. In both these models, blood glucose and insulin concentrations are profoundly elevated demonstrating that the mice are overtly diabetic, a condition known to induce muscle atrophy [13]. Conversely, mice fed a high-fat diet for a shorter duration (2 months or less) have no apparent defects in basal muscle protein metabolism [14], but demonstrate whole-body insulin resistance and impaired muscle mitochondria function [15, 16]. High-fat fed mice also have an impaired ability to increase translation of muscle proteins during load-induced hypertrophy [17] and do not have the typical increased rate of muscle protein synthesis in response to a meal [14] demonstrating that the ability to regulate protein synthesis in response to external cues is impaired. However, whether a short-term high fat diet affects the response of muscle to atrophy signals is unknown.

The purpose of this study was to determine whether rapid weight gain induced by a high-fat diet accelerates muscle atrophy due to disuse (loss of innervation). Given that proper function of mitochondria are required to maintain muscle mass [18, 19], we hypothesize that a hypercaloric diet as characterized by rapid weight gain will make muscles more susceptible to atrophy. To test this, mice were fed a high fat diet for 3 weeks and atrophy induced by surgical sectioning of the sciatic nerve.

## Methods

### Animals and study design

Six-week old, male C57BL/6 mice were purchased from Charles River Laboratories (Raleigh, NC). Mice were housed at 22 °C in a 12:12 h light:dark cycle and provided free access to water and standard chow food. After a two week acclimation period, mice were divided into three groups; and each group (*n* = 18–20) was given free access to a different diet for 12 weeks. The control group (Control) was fed standard rodent chow (Prolab, RMH 3000) containing 14 % calories from fat. The 3-weeks high-fat (3wHF) group was fed the control diet for 9 weeks and then switched to a high-fat diet (Research Diets, D12492) containing 60 % calories from fat for an additional 3 weeks. The third group was fed only the high-fat diet (12wHF). Mice were weighed weekly, and body composition (fat mass and fat-free mass) was measured every third week using an EchoMRI 700 Body Composition Analyzer. All animal procedures were approved by the East Carolina University Animal Care and Use Committee.

Muscle atrophy was induced by surgical, unilateral denervation of the lower hindlimb as done previously [20] at either 5 days or 14 days before the end of each diet (*n* = 9–10 per group). Mice were anesthetized with inhaled 2-3 % isoflurane with oxygen. Hair was removed from the lateral surface of both hindlimbs, and the skin disinfected with povidone-iodine. A small incision (<5 mm) was made on the lateral surface of the hindlimb at approximately mid-femur. The sciatic nerve was exposed via blunt dissection, and a 2 mm section was cut and removed. The skin was then closed with surgical glue (3M Vetbond Tissue Adhesive). Sham surgery was performed on the contralateral limb, leaving the sciatic nerve intact. A subcutaneous dose of Buprenex (0.03 mg/kg) was given as an analgesic.

### Sample collection

Blood was collected from a small incision at the tip of the tail and immediately tested for glucose. Mice were anesthetized by an intraperitoneal injection of ketamine:xylazine (100:10 mg/kg) and then euthanized by cervical dislocation. Whole blood was collected by syringe from the abdominal aorta and allowed to clot on ice. The blood was centrifuged, and serum transferred to a separate tube for storage at −80 °C. It is important to note that samples were collected from fed mice, since fasting induces a rapid increase in the rate of protein degradation and expression of atrophy-related genes [21].

Soleus and extensor digitorum longus (EDL) muscles were removed, blotted dry, weighed and then mounted for measures of protein degradation. These muscles represent extremes of metabolic fiber types in the mouse, with the highly-oxidative soleus containing more than 80 % type I and type IIa fibers and the highly glycolytic EDL having greater that 85 % type IIb fibers [22].

### Blood glucose, serum insulin, and serum myostatin

Whole blood glucose levels were measured using a glucometer (OneTouch Ultra 2). Serum insulin was measured in duplicate using an enzyme-linked immunosorbent assay (ELISA) kit for rat/mouse insulin (EZRMI-13 K, Millipore) as directed by the manufacturer. Serum myostatin was measured in duplicate using an ELISA kit for mouse myostatin (E91653Mu, USCN Life Science Inc.) performed as directed by the manufacturer. Importantly, the duration of muscle denervation, which only effects a small proportion of whole-body muscle mass, had no effect on measures of blood glucose, insulin, or myostatin. Therefore, the data of the two denervation duration groups were combined.

### Protein degradation

Rates of protein degradation were determined by measuring the release of the essential amino acid tyrosine from isolated muscles, based on previous methods [23, 24]. Immediately after weighing, muscles were secured with custom plastic clips [25] at approximately resting length. Muscles were incubated for 30 min in Krebs-Henseleit buffer containing 5 mM glucose and 0.15 mM pyruvate. All buffers were maintained at 37 °C and gassed continuously with 95 % O_2_/5 % CO_2_. The muscles were then blotted and transferred to a new incubation well containing 3 ml Krebs-Henseleit/glucose/pyruvate buffer and 0.5 mM cycloheximide, to inhibit reincorporation of amino acids by protein synthesis. Two hours later, muscles were removed, blotted dry, and frozen in liquid nitrogen. The final incubation media was removed and treated with perchloric acid (PCA) (0.2 N final concentration) to precipitate proteins and small peptides. The PCA soluble tyrosine in the buffer was measured by first derivatizing the samples with Waters AccQTag technology [26] and then quantifying the derivatized amino acids using a Waters Acquity Ultra Performance Liquid Chromatograph H-class. Rates of protein degradation are given as nmol tyrosine per mg muscle per hour.

### Protein analysis

Frozen soleus and EDL muscles were homogenized in ice-cold RIPA buffer and diluted to a final protein concentration of 500–2500 ng/μl in sample buffer (2 % SDS, 80 mM Tris–HCl, 22 % glycerol, 50 mM DTT, bromophenol blue). Protein concentration was determined by bicinchoninic acid assay with bovine serum albumin as the standard (ThermoScientific).

Myosin heavy chain (MHC) isoforms were separated using a mini-gel electrophoresis system (BioRad) as we have done previously in cultured myotubes [27]. Samples (5 μl per well) were loaded and electrophoresis was performed with a 35 % v/v glycerol, 8 % w/v acrylamide-N,N’-methylenebisacrylamide (bis) (9:1) gel for 22 h at a constant 130 volts at 4 °C. The gels were then silver stained (Pierce Silver Stain Kit), and images were captured using a BIO RAD Molecular Imager ChemiDoc™ XRS+ Imaging System and analyzed using Image Lab 3.0 software. Identity of specific MHC isoforms was initially confirmed by immunoblotting.

For the autophagy related protein LC3, ubiquitin, and mitochondrial proteins, equal amounts of total protein were separated by 10 % SDS-PAGE and transferred to polyvinylidene difluoride membranes. Membranes were incubated overnight with a primary antibodies against: mono and poly ubiquitinated conjugates (#BML-PW8810, Enzo), LC3B (#3868, Cell Signaling), CoxIV (#4844, Cell Signaling), or cocktail against proteins in electron transport chain complexes II-V (Mitoprofile Total OXPHOS Rodent, Mitosciences). Secondary antibodies were conjugated to horseradish peroxidase and detected using an enhanced chemiluminescent substrate (Millipore). Band intensities were captured using a Bio-Rad Chemi Doc XRS+ and analyzed using Image Lab 3.0 (BioRad) or ImageJ (NIH) software [28].

### Statistical analysis

All data are expressed as mean ± standard error of the mean. Significant differences (*P* < 0.05) were assessed using two-way ANOVA (for comparisons including both diet and innervation status), one-way ANOVA (for comparisons of only diet groups). If significance was detected by ANOVA, Tukey’s post hoc analysis was used to determine which groups were different. All analyses were performed using GraphPad Prism for Mac, version 6.0 f.

## Results

### Body composition and insulin resistance

**Fig. 1:**
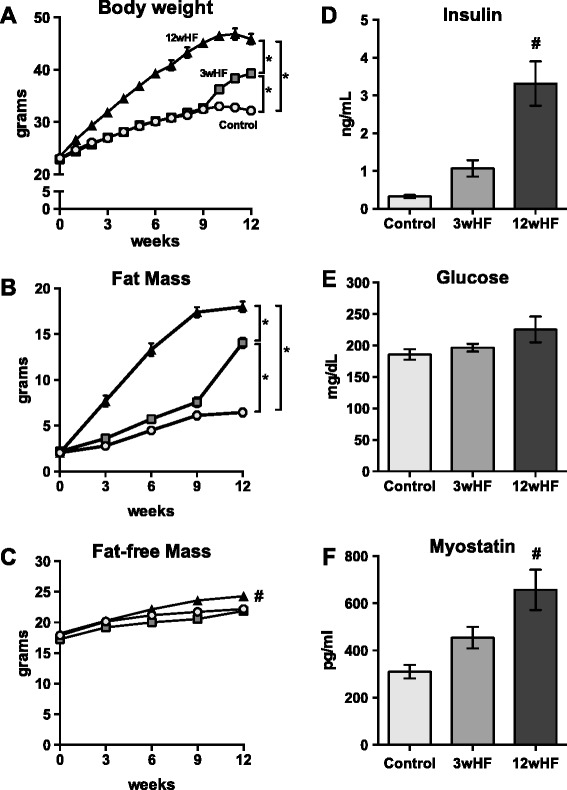
Body composition and blood metabolites of mice on control or high-fat diets. Starting at week 0, mice were kept on the low-fat diet for 12 weeks (control), fed the high-fat for 12 weeks (12wHF), or fed the control diet for 9 weeks followed by the high-fat diet for 3 weeks (3wHF). Body weight (**a**) was measured weekly. Fat mass (**b**) and fat-free mass (**c**) were measured by EchoMRI every 3 weeks. At the completion of the diet, serum insulin (**d**), whole blood glucose (**e**), and serum myostatin (**f**) were measured. *n* = 18–20 per diet; *P < 0.05; #*P* < 0.05 vs control and 3wHF

To establish a model of weight gain without overt diabetes, mice were separated into three dietary groups. Mice were fed for 12 weeks one of the following diets: standard rodent chow (control), high-fat chow (12wHF), or standard chow for 9 weeks followed by a high-fat chow for 3 weeks (3wHF). At the completion of the diets, mice on the 3wHF diet were 22 % heavier and mice on the 12wHF diet were 42 % heavier than the control group (Fig. 1a). The majority of this increase in body weight was due to an increase in the amount of fat. As compared to mice on the control diet, the 3wkHF mice had 220 % more fat, while the 12wHF mice had 280 % more fat (Fig. 1b). Only the 12wHF group gained significantly more fat-free mass (8 %) (Fig. 1c).

Serum insulin and blood glucose were analyzed to estimate insulin resistance, a known complication of obesity [13, 29]. Serum insulin was increased 9.1-fold (*P* < 0.05) by the 12wkHF diet but was not significantly altered with the 3wHF diet (Fig. 1d). However, blood glucose concentration was not significantly different among the diet groups (Fig. 1e).

Myostatin is a muscle secreted protein that negatively regulates muscle mass [30] and increases in severely obese rodents [31] and humans [32]. Serum levels of myostatin of the 3wkHF group were not different than controls, but myostatin concentrations were more than doubled (2.2-fold greater, p ≤ 0.05) in the 12wkHF group (Fig. 1f). Taken together, the 3wHF diet, with continuous weigh gain even through the final week of the diet and modestly, but non-significantly, increased insulin and myostatin levels, establishes a model to test the effects of rapid weight gain in the absence of overt diabetes. Therefore, all subsequent studies were conducted with muscles of the 3wHF mice only.

### Loss of mass of denervated muscles

**Fig. 2:**
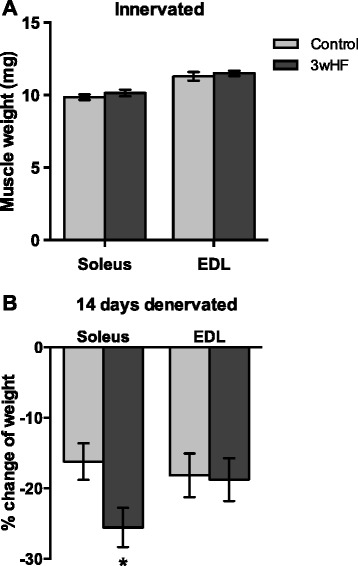
Mass and atrophy of hindlimb muscles. **a** Weights of innervated soleus and extensor digitorum longus (EDL) muscles. **b** Percent change of muscle mass of 14 day denervated muscles = (weight of denervated muscle - weight of contralateral innervated muscle)/wet weight of innervated muscle x 100. *n* = 9–10 per group; *P < 0.05 vs control

Since skeletal muscle is a major component of fat-free mass, differences in fat-free mass may be reflected by differences in muscle mass. To determine whether a high-fat diet increased the weight of specific muscles, individual innervated muscles of the lower hind limbs were removed and weighed. In agreement with fat-free mass (Fig. 1c), the 3wHF diet had no significant effect on the mass of innervated soleus or EDL muscles (Fig. 2a). To induce muscle atrophy, hindlimb muscles were surgically denervated with the contralateral limb serving as the innervated control. Five days later, little atrophy had occurred; the denervated muscles weighed within 5 % of the contralateral innervated muscles regardless of the diet (data not shown). However, in the control-fed mice, fourteen days denervation induced 16 % loss of mass in the soleus and 18 % loss of mass of the EDL (Fig. 2b). Interestingly, the 3wkHF diet substantially increased the amount of atrophy only in the soleus. The soleus muscles of the 3wkHF group lost 26 % of their mass (Fig. 2b).

### High-fat diet increases protein degradation rate in the soleus

**Fig. 3:**
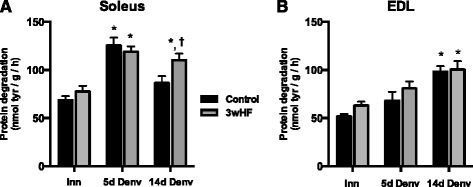
Muscle protein degradation rates of mice fed a high-fat diet for three or twelve weeks. Soleus (**a**) and EDL (**b**) muscles were incubated in Krebs-Henseleit buffer containing cycloheximide, and tyrosine release was measured over time. *n* = 9–10 per group; *P < 0.05, vs innerv. †*P* < 0.05 vs control

In lean mice, muscle atrophy due to denervation is mediated mainly by an increase in the rate of protein degradation [7, 33]. To determine whether the increased muscle atrophy in the soleus of the 3wHF was a result of increased proteolysis, we measured protein degradation by the release of the essential amino acid tyrosine. In soleus muscles, the rates of protein degradation increased rapidly in the 5 day denervated muscles to 70 % greater than innervated muscles regardless of the diet. By 14 days post-denervation, the degradation rates of the control fed mice decreased to rates not different from innervated, while rates in the 3wHF mice remained significantly increased (Fig. 3a). In EDL muscles, the rates of protein degradation increased approximately 33 % in 5 day denervated muscles and further increased (68 % or more) in 14 day denervated muscles (Fig. 3b). The high-fat diet had no effect on rates of protein degradation in the EDL.

**Fig. 4:**
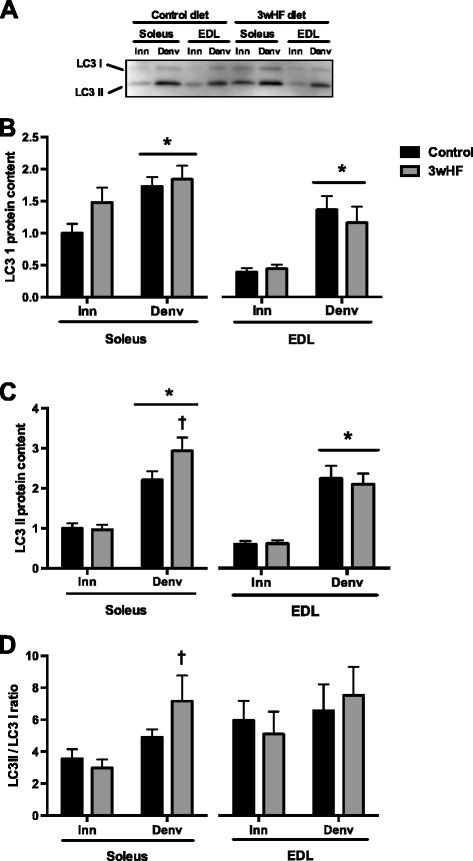
LC3 content is increased by 14 days denervation. Mice were fed a low-fat diet (control) or a high-fat diet for 3 weeks (3wHF). The muscles of one hindlimb were denervated (Denv) for the final 14 days, and the other hindlimb served as the innervated control (Inn). **a** Representative LC3 immunoblot. **b** Relative quantification of LC3 I, **c** LC3 II, and **d** the ratio of the band intensities of LC3 II/LC3 I. *n* = 9–10 per group; *P < 0.05 main effect vs innervated. †*P* < 0.05 vs control

The increased protein degradation of muscle atrophy is mediated largely by activation of the autophagic/lysosomal and ubiquitin/proteasomal pathways [9, 34]. One indicator of the activation of autophagy/lysosomal pathway is the abundance of LC3 [35]. In mammalian cells LC3 is predominantly in two forms: LC3-I, which is cytosolic, and LC3-II, which is associated with autophagosomes (Fig. 4a). In the soleus and EDL muscles, both LC3-I (Fig. 4b) and LC3-II (Fig. 4c) were more abundant in the denervated than in the innervated muscles. Importantly, the 3wHF diet induced LC3-II further (*P* < 0.05) in the denervated soleus muscle leading to an increase in the LC3-I/LC3-II ratio (Fig. 4d). However, in the EDL, the 3wHF diet had no significant effect on LC3-I abundance (Fig. 4b), LC3-II abundance (Fig. 4c), or the ratio of LC3-I/LC3-II (Fig. 4d).

**Fig. 5:**
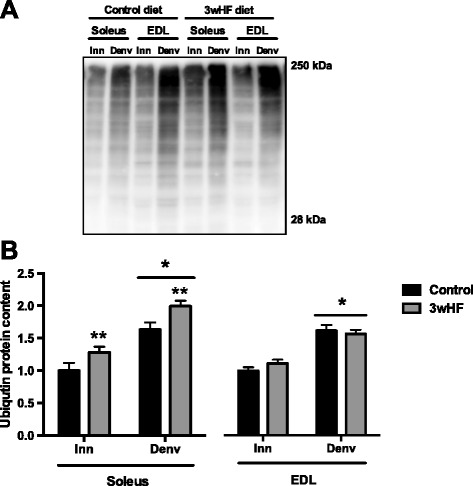
Ubiquitinated conjugates accumulate by 14 days denervation. Mice were fed a low-fat diet (control) or a high-fat diet for 3 weeks (3wHF). The muscles of one hindlimb were denervated (Denv) for the final 14 days, and the other hindlimb served as the innervated control (Inn). **a** Representative ubiquitin immunoblot. **b** Relative quantification band intensities. *n* = 9–10 per group; *P < 0.05 main effect vs innervated. †*P* < 0.05 vs control

Proteins are targeted for degradation by the proteasome by being labeled by ubiquitin chains. Therefore, as an indicator of upregulation of the ubiquitin/proteasome pathway, we next examined the amount of ubiquitin-protein conjugates. Denervation increased the amount of ubiquitination in both the soleus and EDL (Fig. 5b). Furthermore, 3wHF diet had a main effect to increase ubiquitination in the soleus but had no effect in the EDL (Fig. 5b)

### Myosin heavy chain and mitochondrial proteins

**Fig. 6:**
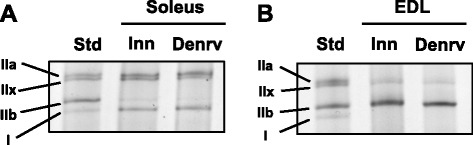
Representative images of myosin heavy chain isoform separation by high-resolution electrophoresis. Soleus (**a**) and EDL (**b**) muscles that were denervated for 14 days (Denrv) or innervated (Inn) were homogenized, and equal amounts of total protein were separated by high-resolution electrophoresis. Proteins were silver stained for visualization

Muscle atrophy varies among skeletal muscles with different fiber types [36], and fiber type may change in response to a high-fat diet [37] or denervation [38]. Therefore, differences in muscle mass loss may be explained by changes in fiber type. As an indicator of muscle type, the percent of myosin heavy chain (MHC) isoforms was determined by high-resolution electrophoresis and silver staining of homogenates of the soleus (Fig. 6a) and EDL (Fig. 6b). The soleus muscles expressed an abundance (>75 %) of type I and type IIa MHC (Table 1). Fourteen days of denervation had a main effect to increase the percent of type I MHC protein and decrease type IIa. EDL muscles expressed predominantly (>85 %) fast-twitch type IIb MHC (Table 2). Fourteen days of denervation had a main effect to increase the percent type IIa fibers and decrease the percent IIb fibers, i.e. a shift away from the fastest fiber-type. The 3wHF diet had no significant effect on MHC isoform expression either in the soleus or EDL.

**Table 1:** Percent MHC isoform protein of innervated (Inn) or 14 day denervated (Denv) soleus muscles

	Type I		Type IIa		Type IIx		Type IIb	
	Inn	Denv	Inn	Denv	Inn	Denv	Inn	Denv
Control	29.6 ± 1.3	41.7 ± 2.4*	50.3 ± 2.2	40.1 ± 2.3*	15.7 ± 1.9	12.4 ± 1.6	4.5 ± 1.6	5.9 ± 3.5
3wHF	30.7 ± 1.4	40.9 ± 2.2*	47.9 ± 1.8	41.3 ± 1.6*	15.3 ± 1.5	10.0 ± 1.2	6.1 ± 1.9	7.8 ± 2.7

*P < 0.05 vs Inn

**Table 2:** Percent MHC isoform protein of innervated (Inn) or 2 week denervated (Denv) EDL muscles

	Type I		Type IIa		Type IIx		Type IIb	
	Inn	Denv	Inn	Denv	Inn	Denv	Inn	Denv
Control	n.d.	n.d.	13.4 ± 0.9	17.1 ± 1.4	n.d.	3.6 ± 1.6	86.6 ± 0.9	79.3 ± 1.0*
3wHF	n.d.	n.d.	12.4 ± 1.0	22.5 ± 2.3*	n.d.	n.d.	87.6 ± 1.0	77.5 ± 2.3*

n.d. = none detected. *P < 0.05 vs Inn

**Fig. 7:**
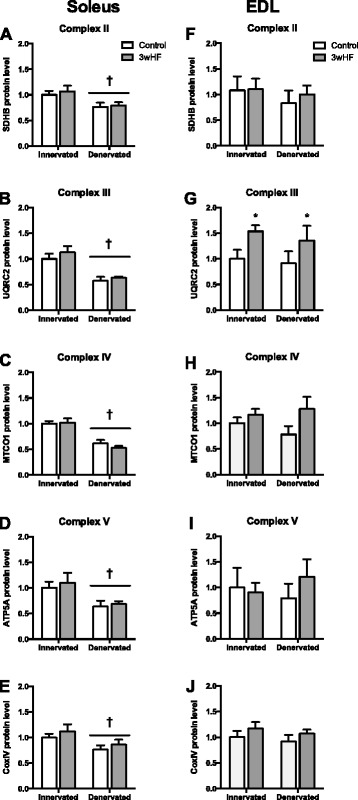
Mitochondrial protein expression decreases with 14 day denervation in the soleus but not in the EDL. Immunoblot analysis for several mitochondrial proteins was performed on the soleus (**a**-**e**) and EDL (**f**-**j**) to determine the response to denervation and a high-fat diet. Data normalized to control diet, innervated muscle. *P < 0.05 main effect vs Innerv

Aside from myosin heavy chain, different muscles are characterized by different metabolic properties [39]. Therefore, we also measured the amount of several mitochondrial electron transport chain proteins as indicators of mitochondrial content. In the soleus, 14 days denervation decreased the protein amount of all mitochondrial proteins measured: ATP5A, SDHB, UQRC2, MTCO1, and CoxIV (Fig. 7a-e). The 3wHF diet had no effect on these proteins in the innervated or denervated muscles.

In EDL muscles, the abundance of mitochondrial proteins was not significantly different between the denervated and innervated muscle (Fig. 7f-j). The 3wHF diet had a significant effect on only one protein, UQRC2, which is part of the electron transport chain complex III. UQRC2 increased in both the innervated and denervated EDL muscle in response to the 3wHF. Taken together, the pattern of response differs substantially between the soleus and EDL muscles, with 14 days denervation inducing loss of mitochondrial proteins only in the soleus.

## Discussion

The major finding of this study is that a short-term high-fat diet accelerates skeletal muscle atrophy in the primarily oxidative soleus muscle. A high-fat diet for three weeks, which has little effect on basal muscle size and rates of protein degradation, augments denervation-induced loss of mass, induction of rates of protein degradation, accumulation of the autophagosome marker LC3, and accumulation of ubiquitinated proteins in the soleus. The high-fat diet had no effect on these parameters in the EDL muscle. Therefore, a high-fat diet enhances the activation of protein degradation in the predominantly oxidative fibers of the soleus muscle.

Muscle type-specific differences in protein degradation and atrophy demonstrated here due to a hypercaloric diet are consistent with muscle type-specific responses to various atrophy signals. For instance, Duchenne muscular dystrophy [40], fasting [41], and glucocorticoid administration [42] preferentially affect fast skeletal muscle fibers. On the other hand, muscles composed of a substantial proportion of slow fibers (e.g. rodent soleus) are often, but not always, more susceptible to inactivity or spinal cord injury [43]. The soleus is phenotypically unique among mouse lower hindlimb muscles in that it has a substantial proportion of slow twitch, oxidative type I fibers and few to no fast-twitch glycolytic type IIb fibers [22, 44]. The other muscle examined herein, the EDL, has little to no MHC type I fibers and a substantial amount of type IIb fibers [22, 44]. The mouse soleus muscle also has greater oxidative capacity as evidence by having greater activity of several mitochondrial enzymes [45] and greater coupled and uncoupled respiration [46] as compared to the EDL. Therefore, our findings suggest that high-fat feeding preferentially induces the susceptibility to enhanced rates of protein degradation and loss of mass in the highly oxidative, type I skeletal muscle fibers.

Along with myosin heavy chain and mitochondrial proteins, a large number of other proteins are differentially expressed among different muscle fiber types of the mouse [39, 47], any of which may be critical for the differential response shown here to the high-fat diet. A promising explanation for a soleus-specific response is the transcriptional coactivator PGC-1α, which is a potent stimulator of mitochondrial synthesis. PGC-1α is more highly-expressed in the soleus than in other hindlimb muscles [48], and its mRNA and protein levels decrease in most, if not all, atrophy conditions [21, 49]. On the other hand, PGC-1α protein, but not mRNA, levels increase following 4 weeks or more of a high-fat diet [50]. Importantly, PGC-1α inhibits muscle protein degradation and disuse atrophy, and this inhibition can be disassociated from mitochondrial content [20, 51]. Therefore, a pronounced decrease in PGC-1α expression or intracellular sequestration to keep PGC-1α out of the nucleus would accelerate protein degradation and enhance atrophy. To determine the role of PGC-1α in high-fat diet accelerated atrophy, future studies would be required that determine the muscle fiber-type specific PGC-1α expression pattern, intracellular localization, and manipulation of PGC-1α expression in a muscle specific manner during different diet conditions, perhaps by electroporation of overexpression constructs, as done previously [20].

Alternatively, another possible explanation for the increased protein degradation in the soleus is that the 3wHF diet may impart a metabolic defect, perhaps by lipid induced mitochondrial stress [52], that is compounded by atrophy. Mitochondrial defects sufficient to impair energetics would activate the low-energy sensing molecule AMP activated protein kinase, which has been shown to activate protein degradation and lead to atrophy [53], perhaps by activating FoxO [54]. Even modest mitochondrial mutations, insufficient to change oxidative capacity, induce profound mRNA transcript changes [55]. In support of a metabolic defect induced by a short-term high-fat diet, a 5 week but not a 10 week high-fat diet impairs state 3 respiration and ATP synthesis in permeabilized soleus fibers of C57BL/6 mice [15], while a 6 week HF diet impairs state 3 respiration and ATP synthesis in permeabilized fibers of rat soleus but not TA muscles [16]. Importantly, any mitochondrial defect in the soleus imparted by the high-fat diet would be exacerbated on the whole tissue level by denervation atrophy, because the soleus, but not the EDL, have reduced content of mitochondrial proteins.

The ability of a short-term high-fat diet to foster high rates of protein degradation during disuse atrophy may have important clinical implications. For instance, muscle disuse during bed rest, which occurs during hospital stays, is a potent stimulus for atrophy, which then leads to greater weakness and fatigue and extends hospital stays further. Humans that gain weight (i.e. are in positive energy balance) during 5 weeks of bed rest lose more muscle mass than those subjects that are more weight stable even though both groups consumed the same macronutrient composed diet [56]. It is unclear whether the increased atrophy is due to greater lipid accumulation or whether it is the mismatch in energy supply and demand. Interestingly, our body weight data demonstrate that the 3wHF mice are in positive energy balance (cf. Fig. 1), and overnutrition, i.e. providing fuel in excess of what mitochondria require for ATP resynthesis, is proposed to lead to mitochondrial dysfunction [52].

Another noteworthy finding is that the time course response to denervation varies markedly between the soleus and EDL. In control fed mice, basal rates of protein degradation are faster in the soleus versus the EDL (cf. Fig. 3), which agrees well with studies in young rats demonstrating protein turnover is faster in the slow-twitch soleus muscles [41, 57, 58]. Denervation induces a rapid increase in proteolysis in the soleus, with a peak at 5 days after denervation (increased ~85 % versus innervated). In the EDL, the increase in proteolysis is delayed, with the peak occurring at 14 days post-denervation (increased ~90 % versus innervated). The rapid induction of proteolysis in the soleus may explain the apparent greater sensitivity of the soleus muscle to inactivity or denervation atrophy [43, 59]. Furthermore, our findings emphasize that fiber-type differences in atrophy may not simply be differences in the magnitude of response, but must also account for differences in the timing of responses.

## Conclusions

In summary our results indicate that a high-fat diet for three weeks induces the rate of protein degradation and increases the amount of disuse atrophy in soleus muscles of mice, but this high-fat diet has no effect on basal protein degradation. This implies that a mismatch between energy supply and energy demand, as evidenced by weight gain during the entire diet, exacerbates the loss of muscle mass when atrophy is activated. Importantly, this increased loss of muscle with the high-fat diet does not appear to be related to circulating glucose or elevated myostatin levels, both known complications of prolonged obesity.
